# Supplementary material for: Outward foreign direct investment and GVC position of manufacturing industry: A perspective on China’s general trade and processing trade structure
Source: PLoS One. 2023 Dec 29;18(12):e0295963. doi: 10.1371/journal.pone.0295963 (PMC10756523; doi:10.1371/journal.pone.0295963)
Supplement: Attachment — Submitted filename: Response to Reviewers.docx [file pone.0295963.s002.docx]

Response to the Editor and Referee

**Title: Outward Foreign Direct Investment and GVC Position of Manufacturing Industry: A Perspective on China's General Trade and Processing Trade Structure**

**Authors: Fei Ren, Dong Le, Ziyu Hu***

Dear Prof :

We really appreciate your response concerning the submission of this paper to *PLOS ONE*. We also would like to express great appreciation to you and the reviewers for these constructive comments. We have thoughtfully taken into these comments. The explanations of what we have revised in response to the reviewers’ comments are given point by point. We hope that all these changes fulfill your requirements as well as the reviewer. Correspondence and calls about this paper should be directed to author at the following address and e-mail. Thanks very much again for your attention to our paper.

Once again, thank you for your time and help to our paper processing.

Yours Sincerely,

Ziyu Hu

Address: Economics School, Zhongnan University of Economics and Law, Wuhan 430073, China;

E-mail:994138246@qq.com

For your guidance, itemized responses to reviewers’ comments are appended below.

**1. Overall**

Firstly, we are thankful to the reviewer for the highly instructive comments and valuable suggestions, which have been considered in this revision. Secondly, we rectified the grammatical inaccuracies included in the previous manuscript. Furthermore, certain sentences were simplified without compromising their original significance. Thirdly, we confirmed that there are no withdrawals in the references and adjusted the formatting of the references to conform to the journal requirements. Finally, we have added a title page in the manuscript to indicate author information, and referred to Table 4 in the main text of manuscript.

**2. Detailed responses**

Thank you very much for reviewing the revised manuscript. We believe that your further comments and suggestions are highly constructive and very helpful for our paper. We have thoughtfully taken into these comments and responded to your constructive suggestions from point by point outlined below. We hope we have addressed all of your concerns.

For the sake of presentation, the comments of the reviewers are reproduced in *italics*, and our responses are given in plain.

**Comments from the reviewers**

Reviewer #1: Review comments

***Comments to the Author***

*“1.* *Now all ok for the structure and discussions, only one point:Some sentences are too long, so it is better to make it shorter, eg the following is too long:*

*This paper analyzes the impact and mechanism of outward FDI on the GVC position of China's manufacturing industry from the perspective of a typical binary trade structure, based on the calculation of the GVC position of China's manufacturing industry under general trade and processing trade from 2003 to 2018.”*

**Response**

We appreciate the insightful feedback you have provided. The grammatical faults present in the initial manuscript have been rectified, and certain sentences have been shortened to enhance the clarity and simplicity of this paper. The revised sentence we have highlighted in red in the original manuscript. For illustrative purposes, the modified abstract is presented hereafter:

**Abstract:** Depending on the trading modes, the effect of Outward Foreign Direct Investment (OFDI) on the manufacturing industry's position within the global value chain (GVC) may differ considerably. This paper examines the GVC position of China's manufacturing industry from 2003 to 2018, specifically focusing on the general trade and processing trade. Drawing upon this premise, this paper analyzes the effect and mechanism by which outward FDI influences the GVC position of China's manufacturing industry. The result demonstrates that: (1) China's processing trade manufacturing industry has a much lower GVC position than general trade manufacturing industry. The GVC position of China's general trade manufacturing industry rose from 2.76 to 2.90 from 2003 to 2018, while the processing trade manufacturing industry remained around 1.93. (2) Outward FDI boosts the GVC position of general trade manufacturing industry through facilitating reverse technology spillover, inducing industry structure upgrading, and enabling export scale expansion. (3) Outward FDI hinders the GVC position growth of processing trade manufacturing industry. The research findings offer theoretical backing for China to develop outward FDI strategies that are tailored to different trading modes within the new framework of dual circulation. These strategies aim to facilitate the transformation and advancement of the manufacturing industry, as well as the growth of the GVC position.

**Comments from the reviewers**

Reviewer #2: Review comments

***Comments to the Author***

*“1. Thank you for your response and improvements.*

*Abstract*

*‘There is a lack on explaining the results which only shows that the GVC position of China's processing trade manufacturing 11 industry is far lower than that of the general trade manufacturing industry. A more specific is needed to highlight any figures or trends that revealed such results.’*

*The author have added specific and improved the abstract with the trend and additional information.”*

**Response**

We sincerely thank the reviewer’s interest and affirmation to our research, and we are also grateful for the reviewer’s instructive comments.

*“2. Introduction and literature review*

*• ‘However, some important elements that have been highlighted in the paper were not been stressed in the introduction such as the elements of reverse technology spillover, structure upgrading and export scale expansion.’*

*Author improved and make justification to several elements: reverse technology spillover, structure upgrading and export scale expansion.*

*• ‘Why the paper used the binary trade structure?’*

*Author improved but still not justify as the link and explanation on specific binary trade still not clear*

*• ‘Elaborate further the world input-output model. How this model is in line to extract that results in this study?’*

*Author did extends the discussion on input-output model and how its contributed in the study.”*

**Response**

Thank you very much to the reviewer for recognizing our last revision. To provide an explanation for our utilization of the binary trade structure, we conducted additional analysis on the distinction between general trade manufacturing and processing trade manufacturing in response to the second concern you raised. The relevant analysis is shown below:

It is noteworthy to mention that the manufacturing industry in China has a substantial size and is characterized by a dual trade structure [10-11]. The dual trade structure primarily encompasses two dominant modes of trade, namely general trade and processing trade. The above two have differences in specific connotation. General trade refers to enterprises in China with import and export operation rights, engaging in unilateral import or unilateral export trade activities. Processing trade refers to the operations of enterprises that engage in the importation (or exportation) of raw and auxiliary materials, parts and components, components, packaging materials, and other materials. These materials are then processed or assembled, resulting in the production of finished goods that are then re-exported (or re-imported). According to China Customs figures, the average proportion of China's processing trade exports between 2002 and 2007 was recorded at 53.07%. Despite experiencing a gradual decline in recent years, this proportion remains as high as 20.1% in 2022. China's import and export commerce exhibits a distinct dual trade structure.

In the realm of manufacturing, notable distinctions exist between general trade and processing trade, particularly with regard to technological sophistication and profitability [12]. The general trade manufacturing industry exhibits a more intricate production chain, hence enhancing its productivity, capacity to absorb technology, and worldwide competitiveness. Conversely, the processing trade manufacturing industry is distinguished by the principles of "both ends out" and "large import, large export". The phrase "both ends out" refers to the scenario when the procurement of raw materials and the distribution of finished products occur in the global market. "Large import" refers to the raw materials required for the production of enterprises, mainly dependent on imports. "Large exports" means that enterprises' large-scale exports are based on large imports. It is evident that the aforementioned features pertaining to the processing trade manufacturing industry contribute to a diminished technological content in production and value added in trade [13-15].

Based on our analysis, it can be inferred that the differences between general trade manufacturing and processing trade manufacturing potentially contributes to a more pronounced divergence in the China's GVC position. This could also cause huge variances in OFDI's impact on China's manufacturing industry's GVC position under different trading forms. Therefore, understanding how OFDI affects the GVC position of general trade manufacturing industry and processing trade manufacturing industry would help China restructure and grow its manufacturing sector industry.

*“3. Research methodology*

*• ‘The author needs source on a statement highlighted about ‘China Science and Technology Statistical Yearbook’.’*

*Author did additional explanation and have added footnote to emphasize the source and explanation of the data.*

*• ‘As for the mechanism analysis, author needs to add evidences of similarities or differences from this analysis against previous studies.’*

*Author has added the explanation*

*• ‘Interestingly, processing manufacturing seems to have no changes significantly. Explain.’*

*Author did additional explanation with three perspectives which are acceptable.”*

**Response**

We would like to thank the reviewer for the comments and evaluations. We appreciate your acknowledgement of the revised paper.

*“4.* *Conclusion*

*‘Conclusion is found to be weak and has less aspect of generalization on the impact and contribution of the results in particular to China and the industry. Further a more comprehensive view of the study are needed to conquer the overall picture. Conclusion needs to add the overall results and unique findings that can contribute to the knowledge area.*

*Since the author did the improvements in the conclusion particularly on the generalization and contribution thus the paper has met the standard.”*

**Response**

We are deeply delighted that the revisions we submitted to this paper were approved by the reviewer. We express sincere gratitude for the favorable feedback provided by the reviewer.

**If editor or reviewers have further comments, please inform us in time, and the authors will make improvements as soon as possible as required.**
